# A hybrid biosynthetic-catabolic pathway for norspermidine production

**DOI:** 10.1042/BCJ20240411

**Published:** 2024-09-16

**Authors:** Bin Li, Jue Liang, Margaret A. Phillips, Anthony J. Michael

**Affiliations:** Department of Biochemistry, University of Texas Southwestern Medical Center, Dallas, TX 75390, U.S.A.

**Keywords:** biosynthesis, evolutionary biology, metabolic engineering, polyamines, synthetic biology

## Abstract

The only known pathway for biosynthesis of the polyamine norspermidine starts from aspartate β-semialdehyde to form the diamine 1,3-diaminopropane, which is then converted to norspermidine via a carboxynorspermidine intermediate. This pathway is found primarily in the Vibrionales order of the γ-Proteobacteria. However, norspermidine is also found in other species of bacteria and archaea, and in diverse single-celled eukaryotes, chlorophyte algae and plants that do not encode the known norspermidine biosynthetic pathway. We reasoned that products of polyamine catabolism could be an alternative route to norspermidine production. 1,3-diaminopropane is formed from terminal catabolism of spermine and spermidine, and norspermidine can be formed from catabolism of thermospermine. We found that the single-celled chlorophyte alga *Chlamydomonas reinhardtii* thermospermine synthase (CrACL5) did not aminopropylate exogenously-derived 1,3-diaminopropane efficiently when expressed in *Escherichia coli*. In contrast, it completely converted all *E. coli* native spermidine to thermospermine. Co-expression in *E. coli* of the polyamine oxidase 5 from lycophyte plant *Selaginella lepidophylla* (*SelPAO5*), together with the *CrACL5* thermospermine synthase, converted almost all thermospermine to norspermidine. Although CrACL5 was efficient at aminopropylating norspermidine to form tetraamine norspermine, SelPAO5 oxidizes norspermine back to norspermidine, with the balance of flux being inclined fully to norspermine oxidation. The steady-state polyamine content of *E. coli* co-expressing thermospermine synthase *CrACL5* and polyamine oxidase *SelPAO5* was an almost total replacement of spermidine by norspermidine. We have recapitulated a potential hybrid biosynthetic-catabolic pathway for norspermidine production in *E. coli*, which could explain norspermidine accumulation in species that do not encode the known aspartate β-semialdehyde-dependent pathway.

## Introduction

**Figure 1.**
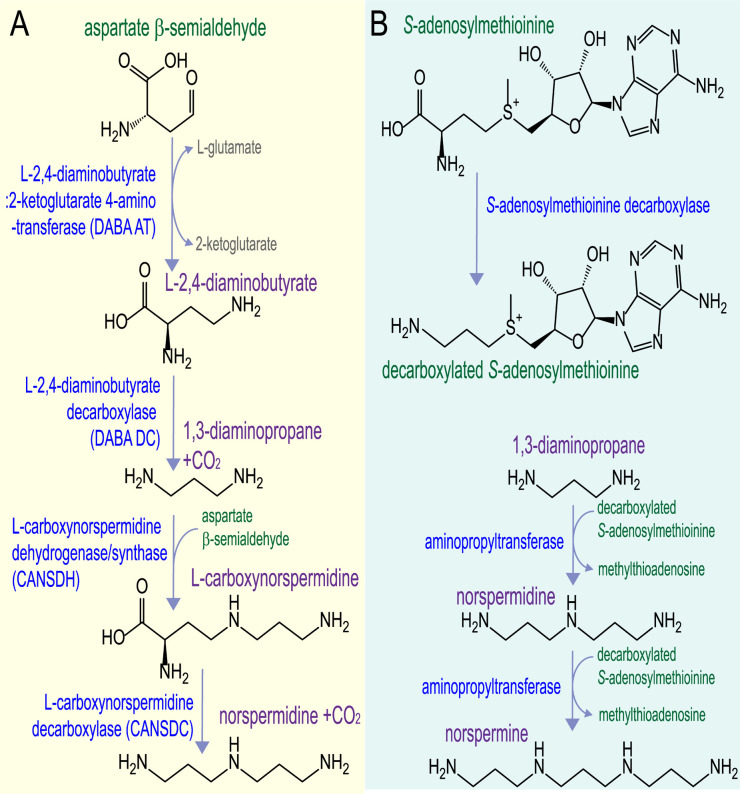
Established and potential pathways for norspermidine biosynthesis. (**A**) The known norspermidine biosynthetic pathway found primarily in the Vibrionales order of the γ-Proteobacteria, based on asparate β-semialdehyde-dependent carboxyaminopropylation of 1,3-diaminopropane. (**B**) A hypothetical pathway for norspermidine and norspermine biosynthesis from 1,3-diaminopropane based on decarboxylated *S*-adenosylmethionine-dependent aminopropylation of 1,3-diaminopropane.

Most eukaryotes, bacteria and archaea produce the triamine spermidine, which is the most prominent polyamine in the biosphere [1]. In eukaryotes and archaea, spermidine is essential for growth and cell proliferation because of its role in the post-translational deoxyhypusine/hypusine modification of translation factor eIF5a/aIF5a [2]. Hypusinated eIF5a is required for correct translation initiation and elongation, and translation of mRNAs encoding polyproline tracts [3]. A less known structural analogue of spermidine is norspermidine, also known as caldine, which is a symmetrical molecule consisting of two aminopropyl groups linked by a central amine (Figure 1). It was discovered initially in a thermophilic bacterium [4] but was subsequently found in bacterial *Vibrio* species. Among *Vibrio* species, norspermidine can form the structural backbone of 2,3-dihydroxybenzoic acid and threonine-based catecholate siderophores including vibriobactin from *Vibrio cholerae* [5], fluvibactin from *Vibrio fluvialis* [6], and vulnibactin from *Vibrio vulnificus* [7]. External norspermidine was found to activate biofilm formation in *V. cholerae* [8–10], and its biosynthesis is essential for biofilm formation [11].

Within the Vibrionales order of the bacterial Pseudomonadota phylum, the biosynthesis of norspermidine (Figure 1A) is dependent on aspartate β-semialdehyde to make the diamine 1,3-diaminopropane using the enzymes l-2,4-diaminobutyrate:2-ketoglutarate 4-aminotransferase (DABA AT), and l-2,4-diaminobutyrate decarboxylase (DABA DC) [12–14]. The 1,3-diaminopropane is then converted to norspermidine by the addition of aspartate β-semialdehyde, i.e. carboxyaminopropylation followed by decarboxylation, achieved by the enzymes l-carboxynorspermidine synthase/dehydrogenase (CANSDH) and l-carboxynorspermidine decarboxylase (CANSDC) [15,16].

However, norspermidine is also found in phylogenetically diverse bacteria, archaea, single-celled eukaryotes, algae and plants [17–23] where the aspartate β-semialdehyde-dependent DABA AT/DC-CANSDH/DC pathway is absent, suggesting the existence of an unknown norspermidine biosynthetic pathway. We hypothesized that norspermidine could be produced by a hybrid anabolic/catabolic pathway that was dependent on decarboxylated *S*-adenosylmethionine to furnish aminopropyl groups, rather than an aspartate β-semialdehyde-dependent pathway. It was possible that 1,3-diaminopropane formed by the terminal oxidation of spermidine [24] could be aminopropylated to form norspermidine (Figure 1B). We found previously that thermospermine synthase from some bacteria can produce norspermidine from 1,3-diaminopropane [25], however, an endogenous source of 1,3-diaminopropane from the associated bacteria was not established. Alternatively, norspermidine could be formed by the biosynthesis of thermospermine from spermidine, followed by oxidation of thermospermine to form norspermidine. A polyamine oxidase (SelPAO5) from the vascular lycophyte plant *Selaginella lepidophylla* has been shown, *in vitro*, to produce norspermidine during the oxidation of thermospermine [22,26]. We generated an *Escherichia coli*-based production platform using heterologous genes to test these hypotheses, and produced a decarboxylated *S*-adenosylmethionine-dependent pathway that converts almost all spermidine in *E. coli* to norspermidine.

## Results

### 1,3-Diaminopropane is unlikely to be the precursor for norspermidine biosynthesis by decarboxylated *S*-adenosylmethionine-dependent aminopropylation

In the absence of the aspartate β-semialdehyde-dependent pathway for 1,3-diaminopropane and norspermidine biosynthesis (Figure 1A), a potential alternative norspermidine biosynthetic pathway is aminopropylation of 1,3-diaminopropane by the decarboxylated *S*-adenosylmethionine-dependent thermospermine synthase (Figure 1B). Thermospermine synthase aminopropylates the terminal 1,3-diaminopropane moiety of spermidine, and previously we showed that phylogenetically diverse bacterial thermospermine synthases are able to produce norspermidine from exogenously supplied 1,3-diaminopropane in *E. coli* [25]. Some plant polyamine oxidases produce 1,3-diaminopropane as a co-product of polyamine terminal catabolism [27,28]. The chlorophyte singled-celled volvocine alga *Chlamydomonas reinhardtii* accumulates at least seven times more norspermidine than spermidine but norspermine is completely absent, as it is in other volvocine algae [21]. We sought to determine whether the *C. reinhardtii* thermospermine synthase (CrACL5) could convert 1,3-diaminopropane to norspermidine when expressed in *E. coli*. As a comparative control, we used the thermospermine synthase of model flowering plant *Arabidopsis thaliana* (AtACL5), which does not accumulate norspermidine. To facilitate the biosynthesis of norspermidine from 1,3-diaminopropane, we expressed the *CrACL5* and *AtACL5* open reading frames (ORFs) from pETDuet-1 in a spermidine-devoid, spermidine synthase gene deletion mutant of *E. coli* BL21 (BL21*speE*).

**Figure 2.**
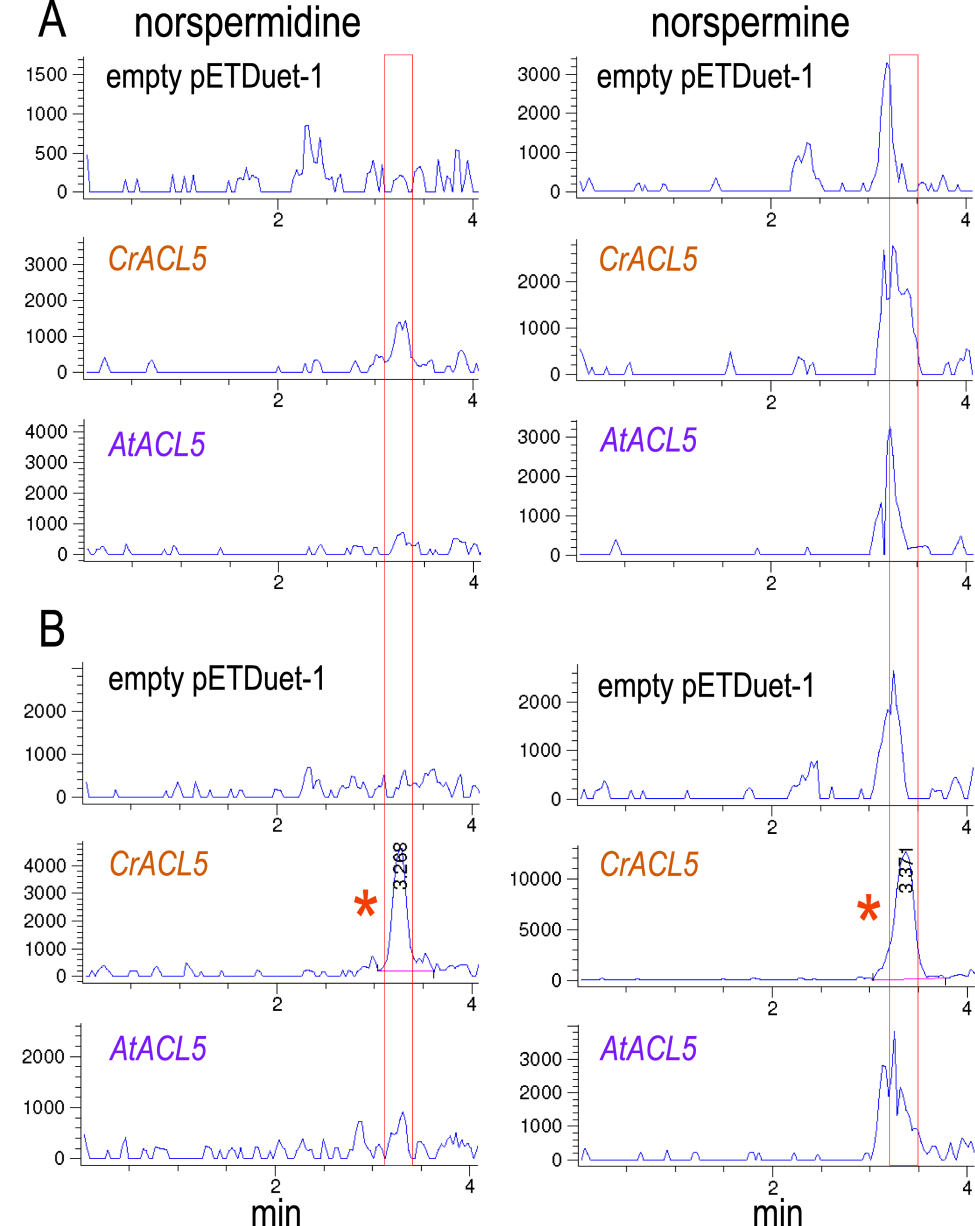
Expression of *Chlamydomonas reinhartii* and *Arabidopsis thaliana* thermospermine synthases in *E. coli* BL21*speE* grown with 1,3-diaminopropane. Extracted Ion Chromatograms (EICs) are shown for tribenzoylated norspermidine (mass window 443.92:444.92) and tetrabenzoylated norspermine (605:606). The *Chlamydomonas reinhardtii CrACL5* (GenBank protein accession number ADF43120) and *Arabidopsis thaliana AtACL5* (acc. no. OAO96167) thermospermine synthases were expressed from pETDuet-1 in spermidine-devoid *E. coli* BL21*speE*, and cells were grown in polyamine-free M9 medium. All samples were grown and analyzed together. The *y*-axis represents arbitrary units of ion intensity. Peaks for norspermidine and norspermine are outlined by the red boxes and where present are indicated by a red asterisk. (**A**) Growth with 0.5 mM 13,-diaminopropane. (**B**) Growth with 5.0 mM 1,3-diaminopropane.

The BL21*speE* strains containing either empty pETDuet-1, or pETDuet-1 expressing *CrACL5* or *AtACL5* were grown in polyamine-free chemically defined M9 medium containing 0.5 or 5.0 mM 1,3-diaminopropane (Figure 2). After induced expression of the *CrACL5* and *AtACL5* ORFs, polyamines were extracted from the BL21*speE* cells, benzoylated, and then analyzed by liquid chromatography-mass spectrometry (LC–MS). Extracted Ion Chromatograms (EICs) for tribenzoylated norspermidine and tetrabenzoylated norspermine are shown in Figure 2. We previously demonstrated that *CrACL5* and *AtACL5* efficiently synthesize norspermine (Figure 1B) from exogenously-derived norspermidine when expressed in BL21*speE* [25], and so the EICs for tetrabenzoylated norspermine are also shown in Figure 2. Expression of either *CrACL5* or *AtACL5* did not result in norspermidine accumulation after growth with 0.5 mM 1,3-diaminopropane (Figures 1A and 2). Some norspermidine and more norspermine was produced by expression of *CrACL5* after growth with 5.0 mM 1,3-diaminopropane, but none was produced by *AtACL5*. Even 0.5 mM 1,3-diaminopropane is a supraphysiological level, and very likely is not physiologically relevant. In *C. reinhardtii*, free 1,3-diaminopropane is present at only 2.1% the level of free spermidine [21], and so considering the inefficient biosynthesis of norspermidine from 1,3-diaminopropane by CrACL5, 1,3-diaminopropane is unlikely to successfully compete with spermidine at the active site of CrACL5 in *C. reinhardtii*. The greater accumulation of norspermine compared with norspermidine by the *C. reinhardtii* thermospermine synthase when expressed in *E. coli* in the presence of 5 mM 1,3-diaminopropane, confirms that this enzyme is primarily a tetraamine rather than triamine synthase. That is, the thermospermine synthase is far more likely to synthesize thermospermine from spermidine, than norspermidine from 1,3-diaminopropane.

### Production of norspermidine or spermidine by oxidation of tetraamines

**Figure 3.**
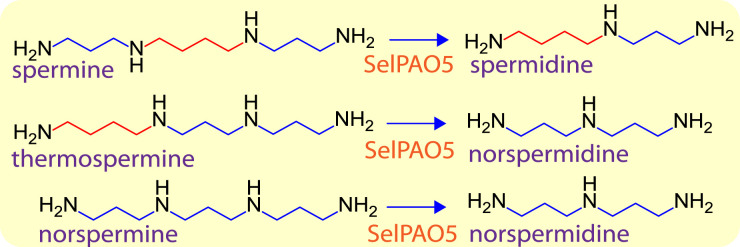
Potential catabolism of tetraamines to triamines by the *Selaginella lepidophylla* polyamine oxidase 5 (SelPAO5) in *E. coli*.

The purified recombinant polyamine oxidase 5 (SelPAO5) of the lycophyte vascular plant *S. lepidopylla* was shown previously, *in vitro*, to oxidize thermospermine to norspermidine, and spermine to spermidine [22]. Expression of the *SelPAO5* ORF in *A. thaliana* polyamine oxidase 5 (*Atpao5-2*) mutant plants that contain thermospermine resulted in the accumulation of a small amount of norspermidine [26]. We sought to determine whether expression of *SelPAO5* in *E. coli* could convert exogenously-derived tetraamines thermospermine, norspermine or spermine to the triamines norspermidine or spermidine (Figure 3). To do this, it was first necessary to establish the purity of commercially obtained thermospermine, spermine, norspermine and norspermidine by LC–MS analysis of the benzoylated polyamines (Supplementary Figure S1). The norspermidine and spermine stocks did not contain any other detectable polyamines, whereas norspermine contained ∼1.5% norspermidine. Our commercially obtained thermospermine contained norspermidine at a level of ∼50% that of the thermospermine, with smaller amounts of spermidine and norspermine. The presence of a high level of norspermidine in the thermospermine stock would confound functional analysis of SelPAO5 with thermospermine as substrate, since SelPAO5 converts thermospermine to norspermidine [22].

As SelPAO5 is also known to convert spermine to spermidine [22], we reasoned that the likely product of SelPAO5 activity with norspermine would be norspermidine. We concluded that SelPAO5 activity with spermine and norspermine could act as a useful indication of SelPAO5 enzymatic functionality in the reducing environment of the *E. coli* cytosol. A spermidine-devoid *E. coli* BL21*speE* strain containing an empty pETDuet-1 plasmid was grown with either 100 µM norspermine, thermospermine or spermine (Supplementary Figure S2). Whereas growth with norspermine or spermine did not result in the accumulation of any detectable norspermidine or spermidine, growth with thermospermine resulted in the accumulation of norspermidine and ∼10% spermidine relative to norspermidine. This indicates that uptake of norspermine or spermine by *E. coli* BL21*speE* does not result in the accumulation of any detectable norspermidine, and so provides a useful system to test SelPAO5 enzymatic activity.

**Figure 4.**
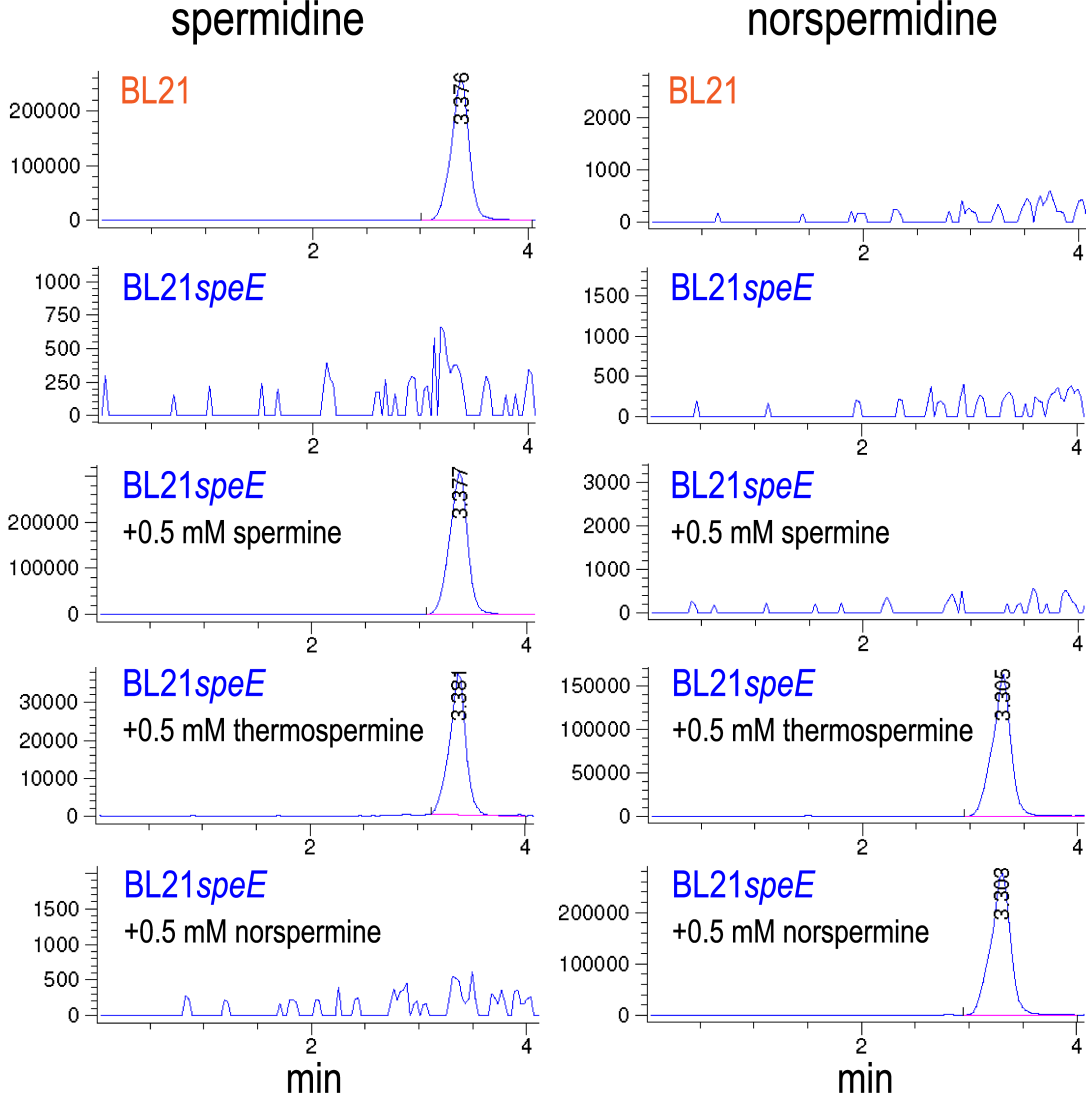
The catabolism of externally-derived tetraamines by expression of *SelPAO5* in spermidine-devoid *E. coli* BL21*speE*. Cultures of *E. coli* BL21*speE* expressing *SelPAO5* (protein acc. no. BAS29669) were grown with either 0.5 mM spermine, thermospermine or norspermine. Shown are the EICs for tribenzoylated spermidine (mass window 457.94:458.94) and tribenzoylated norspermidine (443.92:444.92). The spermidine-replete parental *E. coli* BL21 strain is shown for comparison. All samples were grown and analyzed together. The y-axis represents arbitrary units of ion intensity.

The polyamine oxidase *SelPAO5* was then expressed from pETDuet-1 in BL21*speE* and grown with either 0.5 mM spermine, thermospermine or norspermine (Figure 4). Analysis of benzoylated polyamines revealed that *SelPAO5* expression had efficiently converted spermine to spermidine, and norspermine to norspermidine. The accumulation of spermidine produced from spermine was at a level similar to the native level of spermidine in the BL21 parental strain. These data indicate that the polyamine oxidase SelPAO5 is active within the intracellular reducing environment of *E. coli*.

### Conversion of spermidine to norspermidine via thermospermine

**Figure 5.**
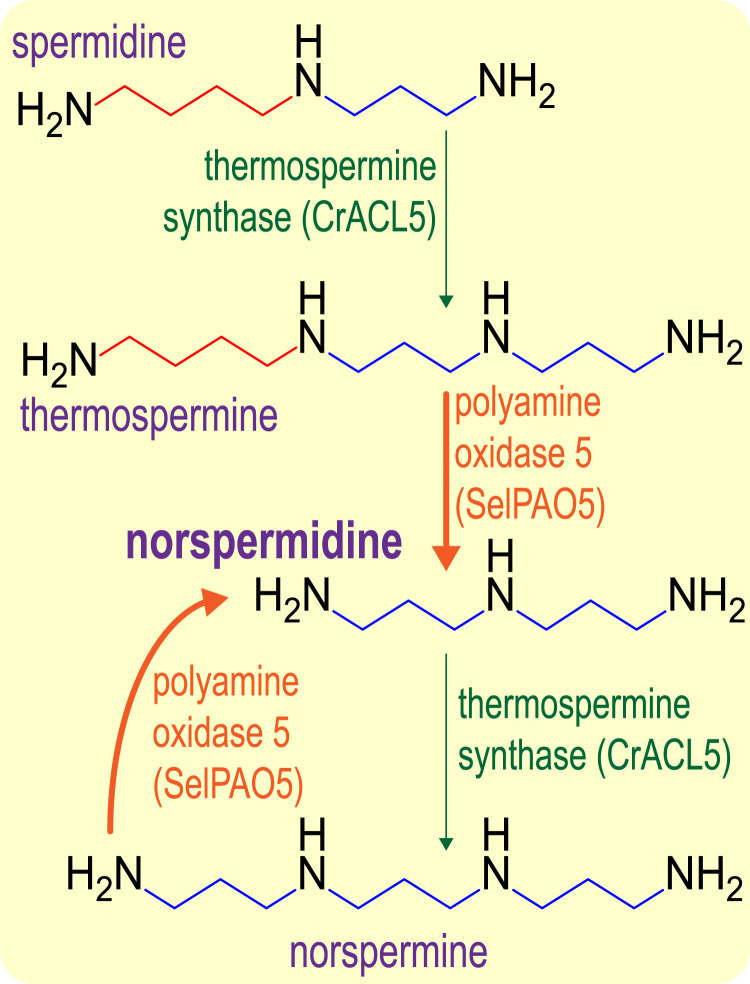
A hypothetical hybrid pathway for norspermidine production.

**Figure 6.**
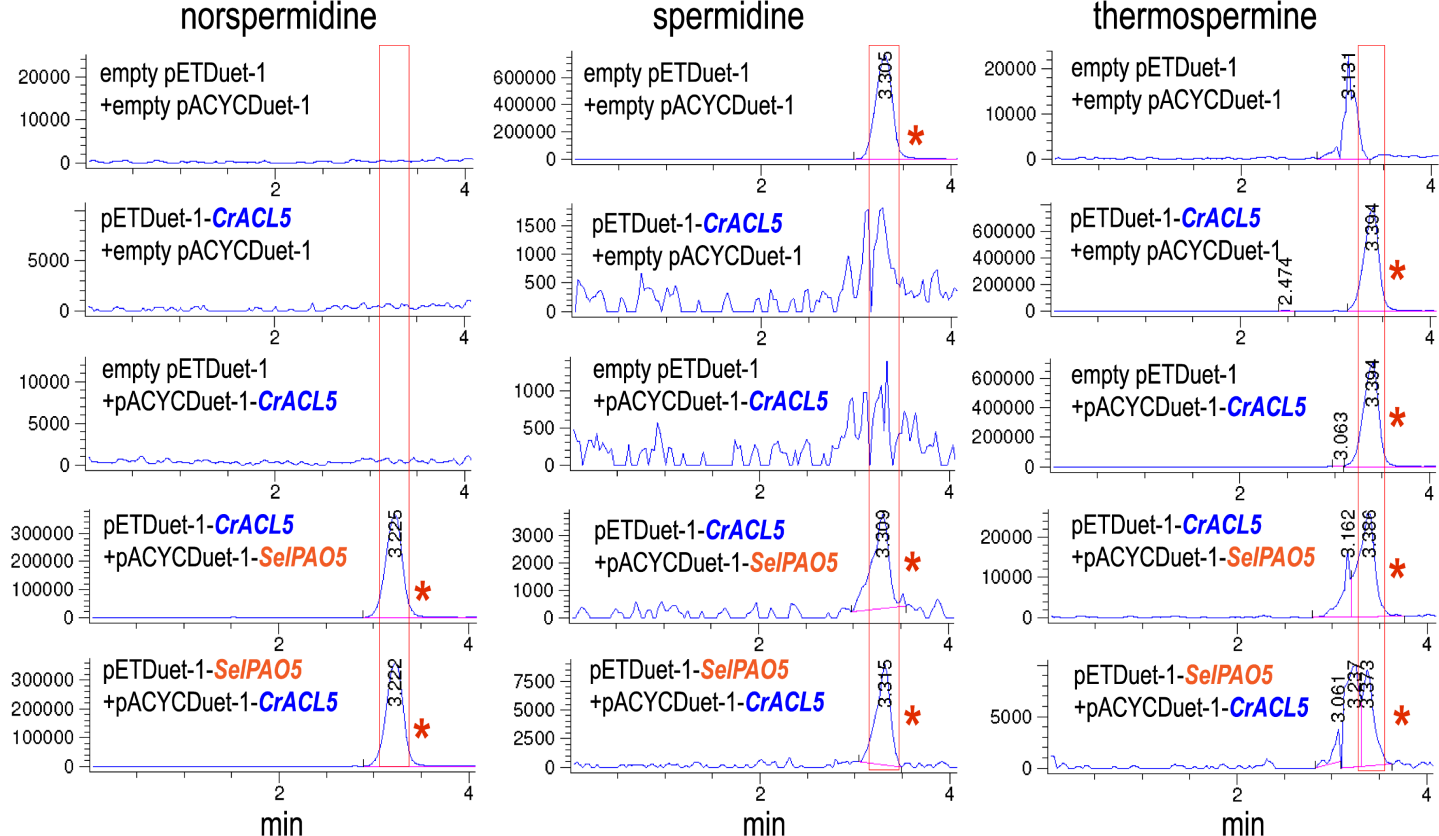
Expression of thermospermine synthase *CrACL5* and polyamine oxidase *SelPAO5* in *E. coli* BL21*speG*. Thermospermine synthase from *Chlamydomonas reinhardtii* (*CrACL5*) and polyamine oxidase 5 from *Selaginella lepidophylla* (*SelPAO5*) genes were expressed in spermidine-replete *E. coli* BL21*speG* from pETDuet-1 or pACYCDuet-1 as indicated, and cell extracts were benzoylated and subjected to analysis by LC–MS. Shown are the EICs for tribenzoylated norspermidine (mass window 443.92:444.92), tribenzoylated spermidine (457.94:458.94) and tetrabenzoylated thermospermine (619.02:620.02). The y-axis represents arbitrary units of ion intensity. The elution positions of polyamines of interest are highlighted by red boxes, and polyamine presence is indicated by red asterisks. All samples were grown and analyzed together.

Expression of *SelPAO5* alone in the spermidine-replete parental *E. coli* BL21 strain produced a trace of 1,3-diaminopropane, <1% of the normal spermidine levels, with no discernible effect on either putrescine or spermidine levels (Supplementary Figure S3). We hypothesized that co-expression of the *C. reinhardtii CrACL5* thermospermine synthase with *SelPAO5* would produce thermospermine from spermidine, that would then be oxidized to norspermidine (Figure 5). In principle, the resulting norspermidine could be aminopropylated by CrACL5 to produce norspermine [25], which might then be oxidized by SelPAO5 back to norspermidine in a futile cycle. To test this hypothesis, we expressed *CrACL5* and *SelPAO5* in spermidine-replete *E. coli* BL21*speG*. In this strain, the spermine/spermidine acetyltransferase *speG* gene is deleted, facilitating accumulation of thermospermine [25]. When *CrACL5* was expressed alone in BL21*speG*, from either the high copy number plasmid pETDuet-1 or the low copy plasmid pACYCDuet-1, all cellular spermidine was completely converted to thermospermine (Figure 6), irrespective of plasmid copy number. The highly efficient activity of CrACL5 in producing thermospermine from spermidine suggests that expression of this gene in *E. coli* would provide a useful synthetic biology platform for thermospermine production.

When *SelPAO5* was co-expressed with *CrACL5*, whether from high or low copy number plasmids, a large amount of norspermidine was produced, and most of the thermospermine disappeared, with the greater accumulation of norspermidine occurring when *CrACL5* was expressed from the high copy number plasmid. Co-expression of *CrACL5* and *SelPAO5* also resulted in the accumulation of a small amount of spermidine, ∼1–2% the level of norspermidine. This may be due to competition between norspermidine and spermidine for the active site of CrACL5, which would spare some of the spermidine from conversion to thermospermine. Co-expression of thermospermine synthase *CrACL5* with polyamine oxidase *SelPAO5* therefore resulted in almost complete conversion of cellular spermidine to norspermidine in *E. coli*. However, no norspermine accumulation was detected by LC–MS. This could be due to oxidation of any norspermine produced back to norspermidine.

### Norspermine produced from norspermidine is dominantly catabolized back to norspermidine

**Figure 7.**
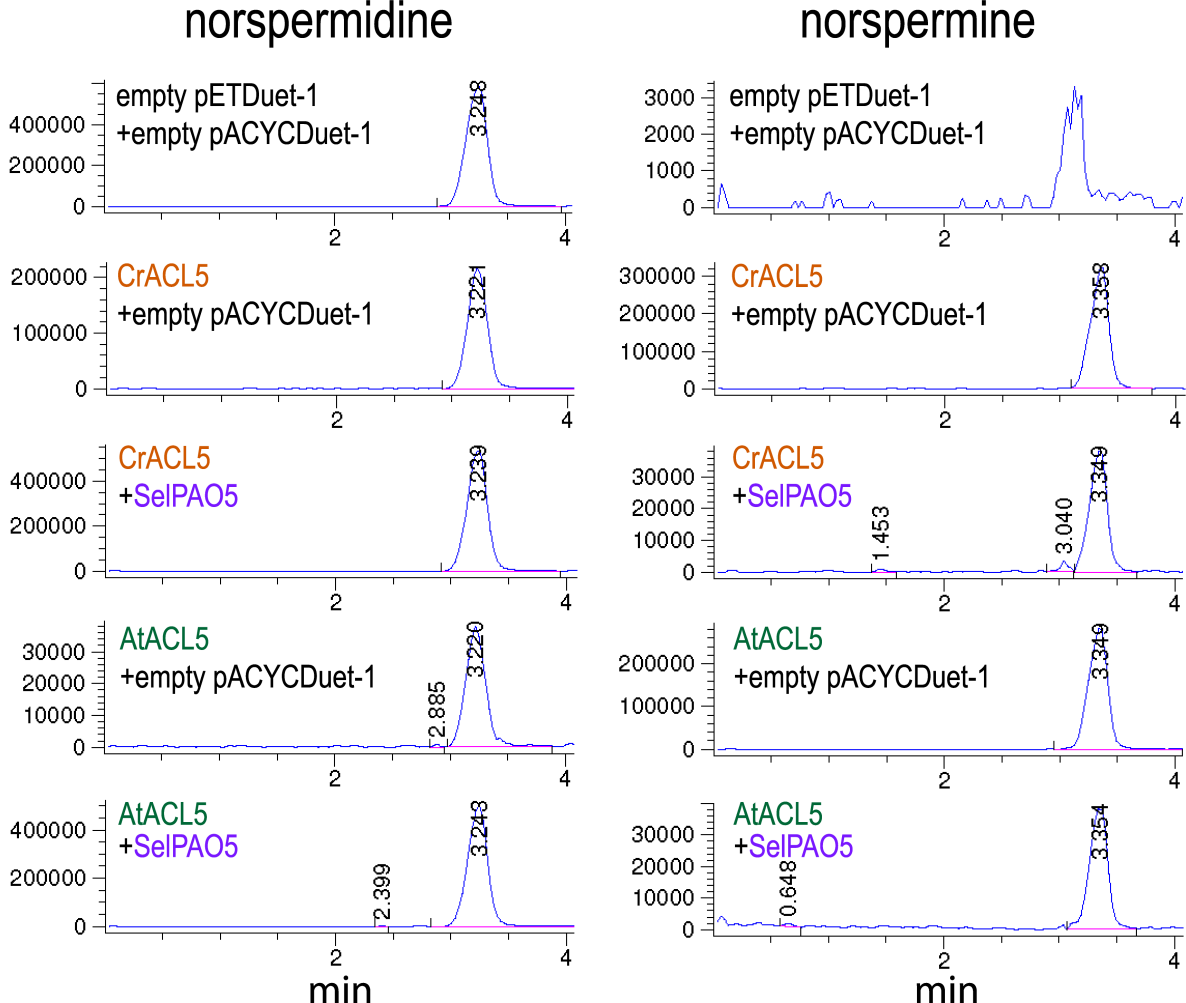
Expression of thermospermine synthases *CrACL5* or *AtACL5* with polyamine oxidase *SelPAO5* in *E. coli* BL21*speE* grown with 1.0 mM norspermidine. Thermospermine synthase from *Chlamydomonas reinhardtii* (*CrACL5*), *Arabidopsis thaliana* (*AtACL5*) and polyamine oxidase 5 from *Selaginella lepidophylla* (*SelPAO5*) genes were expressed in *E. coli* BL21*speE* from pETDuet-1 or pACYCDuet-1 as indicated, and cell extracts were benzoylated and subjected to analysis by LC–MS. Shown are the EICs for tribenzoylated norspermidine (mass window 443.92:444.92) and tetrabenzoylated norspermine (605:606). The *y*-axis represents arbitrary units of ion intensity. All samples were grown and analyzed together.

To assess whether norspermine is produced in this system, and whether it is oxidized back to norspermidine, we expressed *CrACL5*, or the *A. thaliana AtACL5* together with *SelPAO5* in spermidine-devoid *E. coli* BL21*speE* grown with 1.0 mM norspermidine (Figure 7). Norspermine was efficiently produced from accumulated norspermidine by expression of either *CrACL5* or *AtACL5* alone. When either gene was co-expressed with *SelPAO5*, there was an ∼10-fold (*CrACL5*) and 5-fold (*AtACL5*) reduction in norspermine levels, and a 2-fold (*CrACL5*) and 13-fold (*AtACL5*) increase in norspermidine levels, relative to expression of the thermospermine synthases alone. These results suggest that when spermidine is converted to thermospermine, and then efficiently oxidized by SelPAO5 to norspermidine, the resulting norspermidine is aminopropylated by the thermospermine synthase to produce norspermine, and then efficiently oxidized by SelPAO5 back to norspermidine. When *CrACL5* and *SelPAO5* are co-expressed in BL21*speG* (Figure 6), the futile loop between norspermidine and norspermine is driven completely to norspermidine accumulation, with no detectable accumulation of norspermine (Figure 6). In conclusion, the co-expression in *E. coli* of the decarboxylated *S*-adenosylmethionine-dependent biosynthetic *C. reinhardtii* thermospermine synthase *CrACL5* together with the catabolic polyamine oxidase *SelPAO5* from *S. lepidophylla* is a highly efficient system for conversion of spermidine to norspermidine via thermospermine.

## Discussion

The Chlorophyceae class of chlorophyte algae contains single-celled and colonial algae such as *Chlamydomonas*, *Tetrabaena*, *Gonium* and *Volvox*. These algae have been found to contain more norspermidine than spermidine, accumulate only low amounts of thermospermine, and norspermine is absent [20]. At very high concentrations of 1,3-diaminopropane, i.e. non-physiological concentrations, some norspermidine but higher levels of norspermine were produced by the *C. reinhardtii CrACL5* thermospermine synthase expressed in *E. coli*. We concluded that 1,3-diaminopropane is unlikely to be the source of norspermidine biosynthesis by CrACL5 alone because norspermidine biosynthesis from 1,3-diaminopropane is inefficient and norspermine accumulates to a higher level than norspermidine. Norspermine is absent in *C. reinhartii* and other volvocine algae, indicating that a system based solely on thermospermine synthase activity with 1,3-diaminopropane to produce norspermidine is improbable. However, it is possible that an important function of thermospermine in these algae and other organisms, is to allow production of norspermidine via the activity of a thermospermine oxidase. In *C. reinhardtii*, the level of norspermidine is 450-fold higher than spermine/thermospermine [21], which is mostly thermospermine [29]. In our current study, we found that expression of the *C. reinhardtii* thermospermine synthase *CrACL5* in *E. coli* converts all detectable native spermidine to thermospermine. Such an efficient thermospermine synthase would partially explain the large accumulation of norspermidine relative to spermidine and thermospermine in *C. reinhardtii*. It should be emphasized that the functions of thermospermine and norspermidine are not understood in algae, although thermospermine clearly has a major role in vascular development in higher plants.

Thermospermine and norspermidine are also accumulated in the vascular lycophyte plant *S. lepidophylla*, and the recombinant purified polyamine oxidase SelPAO5 is known to oxidize thermospermine to norspermidine *in vitro* [22]. We took advantage of the highly efficient thermospermine synthase of *C. reinhardtii*, together with the known thermospermine oxidase activity of SelPAO5, to recapitulate a potential norspermidine production pathway in *E. coli*. The native polyamines of *E. coli* are the diamines putrescine and cadaverine, and the triamine spermidine. Expression of the *CrACL5* thermospermine synthase alone in *E. coli* resulted in complete conversion of detectable spermidine into thermospermine. When the polyamine oxidase *SelPAO5* was then co-expressed with *CrACL5*, most of the thermospermine produced by CrACL5 was then oxidized to norspermidine, with a trace of spermidine accumulation. The end effect of co-expression of *CrACL5* and *SelPAO5* in *E. coli* was an almost complete conversion of spermidine to norspermidine.

Thermospermine synthase is relatively well conserved across bacteria, archaea and eukaryotes. However, it is likely that the thermospermine oxidase component of this potential norspermidine production pathway will be nonhomologous with SelPAO5 in more phylogenetically diverse organisms such as bacteria or archaea, although the step will be performed probably by an FAD- or FAD/heme-dependent oxidase. This hybrid pathway for norspermidine production consists of two modules, a biosynthetic module to produce thermospermine from spermidine, and a catabolic module to produce norspermidine from thermospermine. The aspartate β-semialdehyde-dependent pathway for norspermidine biosynthesis in *Vibrio* species also consists of two modules: a module for synthesis of 1,3-diaminopropane from aspartate β-semialdehyde, and one for carboxyaminopropylation/decarboxylation of 1,3-diaminopropane to produce norspermidine. These two modules exist separately as single modules in phylogenetically diverse bacteria, for producing 1,3-diaminopropane independently of norspermidine biosynthesis [11], or for producing spermidine from putrescine [30]. The aspartate β-semialdehyde-dependent pathway for norspermidine biosynthesis is therefore itself a hybrid pathway, although it is not clear which module co-opted the other to form the complete norspermidine biosynthetic pathway.

It has been reported [23] that in shoot meristem tissues of the leguminous plant *Medicago sativa*, the tetraamine norspermine is accumulated to higher levels than the triamine norspermidine (Figure 1B). We found that the *CrACL5* thermospermine synthase converts exogenously-derived norspermidine to norspermine in *E. coli*, in the absence of *SelPAO5* expression. In contrast, polyamine oxidase *SelPAO5* expression efficiently converts exogenously-derived norspermine to norspermidine. We did not detect any norspermine accumulation when *CrACL5* and *SelPAO5* were co-expressed in *E. coli*. Similarly, very little thermospermine accumulated after co-expression of *CrACL5* and *SelPAO5*. These findings suggest that the balance of flux is strongly inclined in favor of oxidation such that thermospermine produced from spermidine aminopropylation, and norspermine produced from norspermidine aminopropylation, are efficiently oxidized to norspermidine. The large accumulation of norspermine observed in *M. sativa* likely results from a reversal of this flux direction, either due to lower oxidation activity or from spatial or temporal separation of aminopropylation and oxidation activities.

Although among *Vibrio* species norspermidine is known to participate in siderophore biosynthesis, and to influence biofilm formation and signaling, its function elsewhere is completely unknown. In the single-celled alga *C. reinhartii*, free norspermidine is nearly 10-fold more abundant than spermidine [21], yet its function is a mystery, as it is in all eukaryotes that produce it. This could be an epistemological problem, in that the vital aspect of norspermidine formation from thermospermine may be the formation of co-products hydrogen peroxide and 3-aminopropanal, rather than norspermidine. In plants, it is possible that norspermidine and norspermine may be incorporated into alkaloids. In thermophilic bacteria, norspermidine may be aminopropylated at the secondary amine position to form branched polyamines [31], and norspermine is part of the arsenal of unusual polyamines found in thermophiles that counteract the deleterious effects of growth at high temperature.

The *E. coli* production platform we have developed to engineer norspermidine production could be useful for characterizing potential thermospermine oxidase candidates from other taxa. Production of norspermidine is facilitated by the complete conversion of spermidine to thermospermine by CrACL5. Accumulation of norspermidine after expression of a candidate oxidase would indicate thermospermine oxidase activity. To identify potential spermine oxidase activity, the CrACL5 gene could be swapped for an efficient spermine synthase, such as the human spermine synthase [25]. Spermine oxidase candidates could be expressed in the *E. coli* spermidine-devoid *BL21speE* strain, and production of spermidine would indicate a reverse catabolism activity, whereas production of 1,3-diaminopropane would indicate terminal catabolism. In conclusion, we have demonstrated the activity of a hybrid biosynthetic/catabolic pathway for norspermidine production. It may be the basis of norspermidine production for many phylogenetically diverse species that do not encode the aspartate β-semialdehyde-dependent pathway found in the bacterial Vibrionales order.

## Materials and methods

### Materials

Spermine (cat. No. 85605-1G), norspermine (404810-5G), norspermidine (I1006-100G), spermidine (85580) and 1,3-diaminopropane (D23807-5G) were purchased from Sigma–Aldrich. Thermospermine (Sc-472594B) was purchased from Santa Cruz Biotechnology. Synthetic genes were obtained from GenScript with *E. coli*-optimized codons. Expression plasmids pETDuet-1 and pACYCDuet-1 were obtained from Novagen.

### *Escherichia coli* strains and growth, and gene expression conditions

All employed strains are derived from *E. coli* BL21 (DE3). Construction of BL21*speE* (spermidine synthase gene deletion) and BL21*speG* (spermidine *N*-acetyltransferase gene deletion) was described previously [32,33]. All strains were grown twice in 2 ml of liquid, chemically-defined, polyamine-free M9 minimal medium [34], at 37°C overnight. A 1.0 ml aliquot of culture was then centrifuged, the supernatant discarded, and cells resuspended in 10 ml M9 medium and grown at 37°C to OD_600_ = 0.5. Gene expression from pETDuet-1 and pACYCDuet-1 was induced by addition of 0.2 mM isopropyl-β-d-thiogalactopyranoside and the culture was maintained at 16°C, overnight. Cells were then centrifuged, and the polyamines extracted.

### Polyamine extraction and benzoylation

Cultures of *E. coli* BL21 strains were pelleted by centrifugation, and washed three times by resuspension in PBS. Repelleted cells were resuspended in 200 µl of lysis buffer (100 mM MOPS pH 8.0, 50 mM NaCl, 20 mM MgCl_2_), frozen in liquid nitrogen, thawed at 37°C, and this was repeated three times. To this was added 60 µl of 40% trichloroacetic acid, and after thorough mixing kept on ice for 5 min. Cellular debris was pelleted by centrifugation at 4°C and the supernatant transferred to a new tube for benzoylation, which improves polyamine chromatographic separation and detection. 1,3-Diaminopropane is benzoylated on two amine positions; spermidine and norspermidine on three; spermine, thermospermine and norspermine on four. The mass of the benzoyl moiety is 105 Da. To 200 μl of the cell supernatant containing extracted polyamines, 1 ml of 2 M NaOH was added, then 10 μl of benzoyl chloride, and this mixture was vigorously vortexed for 2 min, and left at room temperature for 1 h. Two ml of saturated NaCl was added to this mixture, followed by further mixing for 2 min, then 2 ml of diethyl ether added, vortexing for another 2 min and left at room temperature for 30 min. The upper layer of diethyl ether containing the polyamines was transferred to a new tube and kept in a chemical hood until fully evaporated.

### Liquid chromatography-mass spectrometry

Samples of benzoylated cell extract were run on an Agilent 1290 Infinity HPLC system fitted with an Eclipse XDB-C18 column (4.6 × 150 mm, 5 µm particle size), coupled to an Agilent 6130 quadrapole ESI mass spectrometer run in positive mode, employing a scan range of 100–1100 m/z. A flow rate of 0.5 ml/min at 20°C was used for the liquid chromatography stage, with a 5 µl injection volume, employing a gradient elution with aqueous acetonitrile containing 0.1% formic acid.

## Data Availability

All relevant data is included in the main text and in the supplementary file.

## Open Access

Open access for this article was enabled by the participation of University of Texas Southwestern Medical Center in an all-inclusive *Read & Publish* agreement with Portland Press and the Biochemical Society under a transformative agreement with Individual.

## Abbreviations

CANSDC: l-carboxynorspermidine decarboxylase
CANSDH: l-carboxynorspermidine synthase/dehydrogenase
DABA AT: l-2,4-diaminobutyrate:2-ketoglutarate 4-aminotransferase
DABA DC: l-2,4-diaminobutyrate decarboxylase
EIC: extracted ion chromatogram
LC–MS: liquid chromatography-mass spectrometry
ORF: open reading frame
