# Supplementary material for: A hybrid biosynthetic-catabolic pathway for norspermidine production
Source: Biochem J. 2024 Sep 16;481(18):1241–53. doi: 10.1042/BCJ20240411 (PMC11531321; doi:10.1042/BCJ20240411)
Supplement: Supplementary Material [file BCJ-481-1241-s1.pdf]

Supporting Information

A Hybrid Biosynthetic-Catabolic Pathway for Norspermidine Production

Bin Li[1], Jue Liang[1], Margaret A. Phillips[1], Anthony J. Michael[1]

[1] *Dept. of Biochemistry, University of Texas Southwestern Medical Center, Dallas, TX 75390, Texas, USA*

Correspondence

Anthony J. Michael, Dept. of Biochemistry, University of Texas Southwestern Medical Center, Dallas, TX 75390, Texas, USA. Email: anthony.michael@utsouthwestern.edu

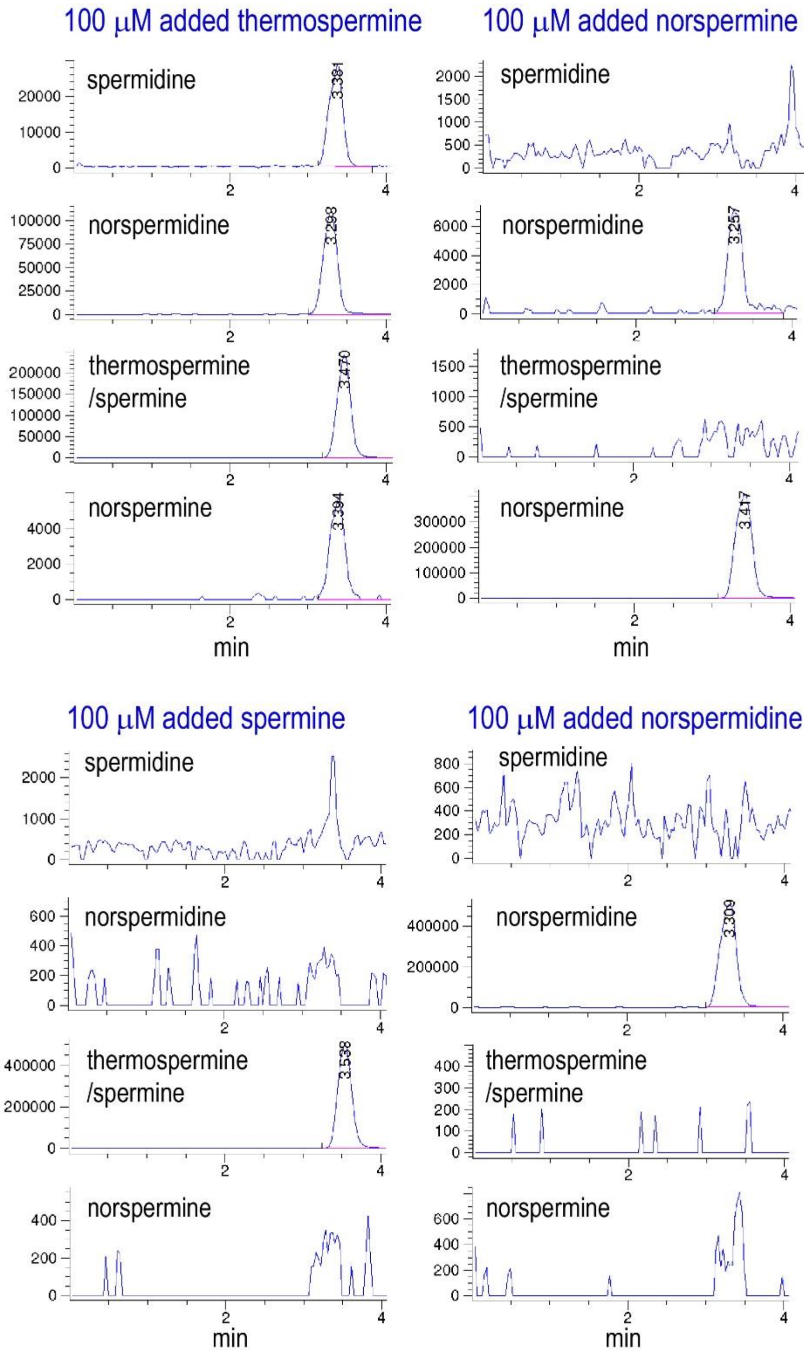

Figure S1. LC-MS analysis of benzoylated aqueous solutions of 100 µM thermospermine, spermine, norspermine and norspermidine. Extracted Ion Chromatograms are shown for tribenzoylated spermidine (mass window 457.94:458.94), tribenzoylated norspermidine (443.92:444.92), tetrabenzoylated thermospermine/spermine (619.02:620.02) and tetrabenzoylated norspermine (605:606). The y-axis represents arbitrary units of ion intensity. All samples were analyzed together.

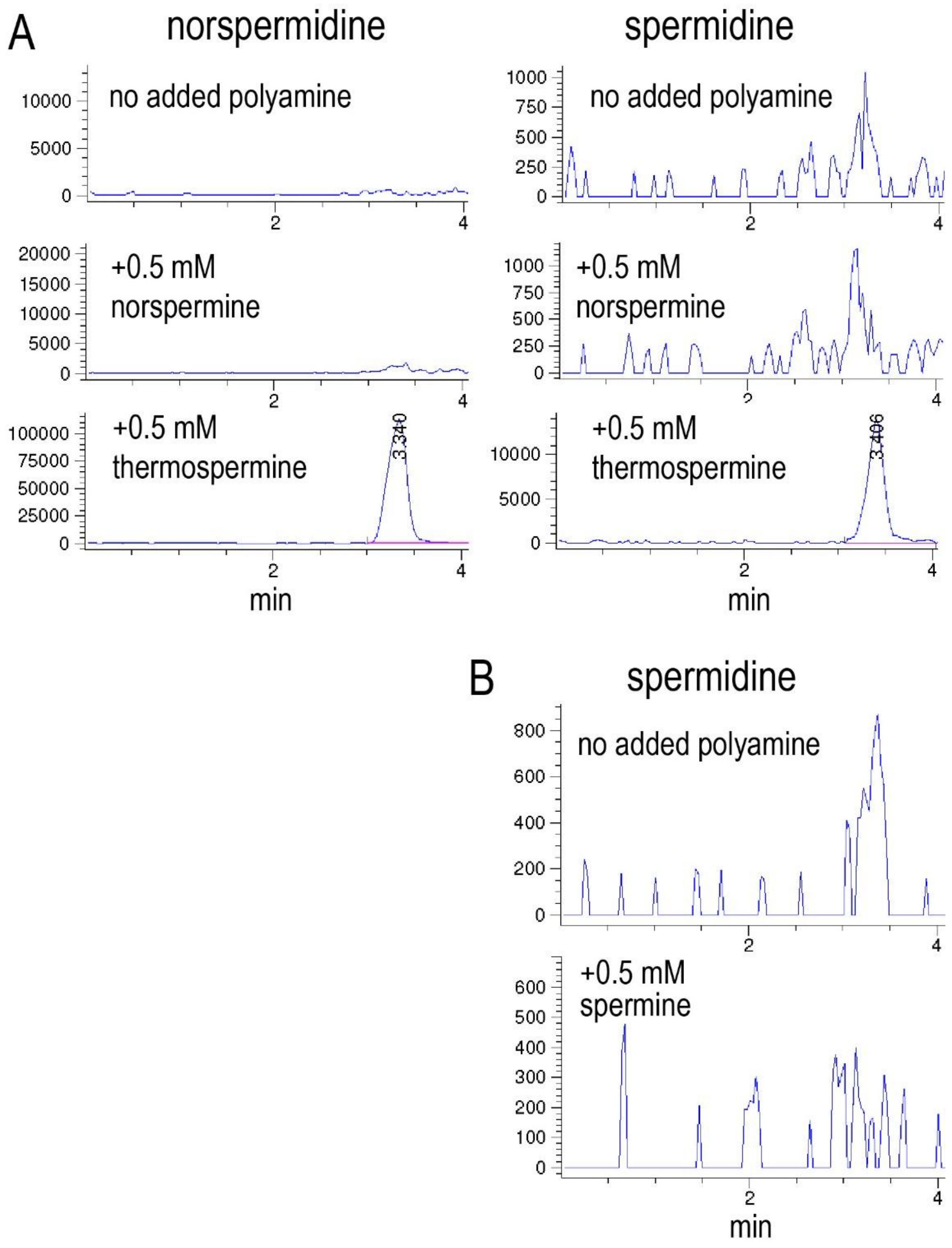

Figure S2. Uptake of thermospermine, norspermine or spermine by spermidine-devoid *E. coli* BL21*speE*. Cells were grown with (**A**) 0.5 mM thermospermine or norspermine, or with (**B**) 0.5 mM spermine. Shown are the Extracted Ion Chromatograms for tribenzoylated spermidine (mass window 457.94:458.94) and tribenzoylated norspermidine (443.92:444.92). The y-axis represents arbitrary units of ion intensity. Samples in (**A**) were grown and analyzed independently of (**B**).

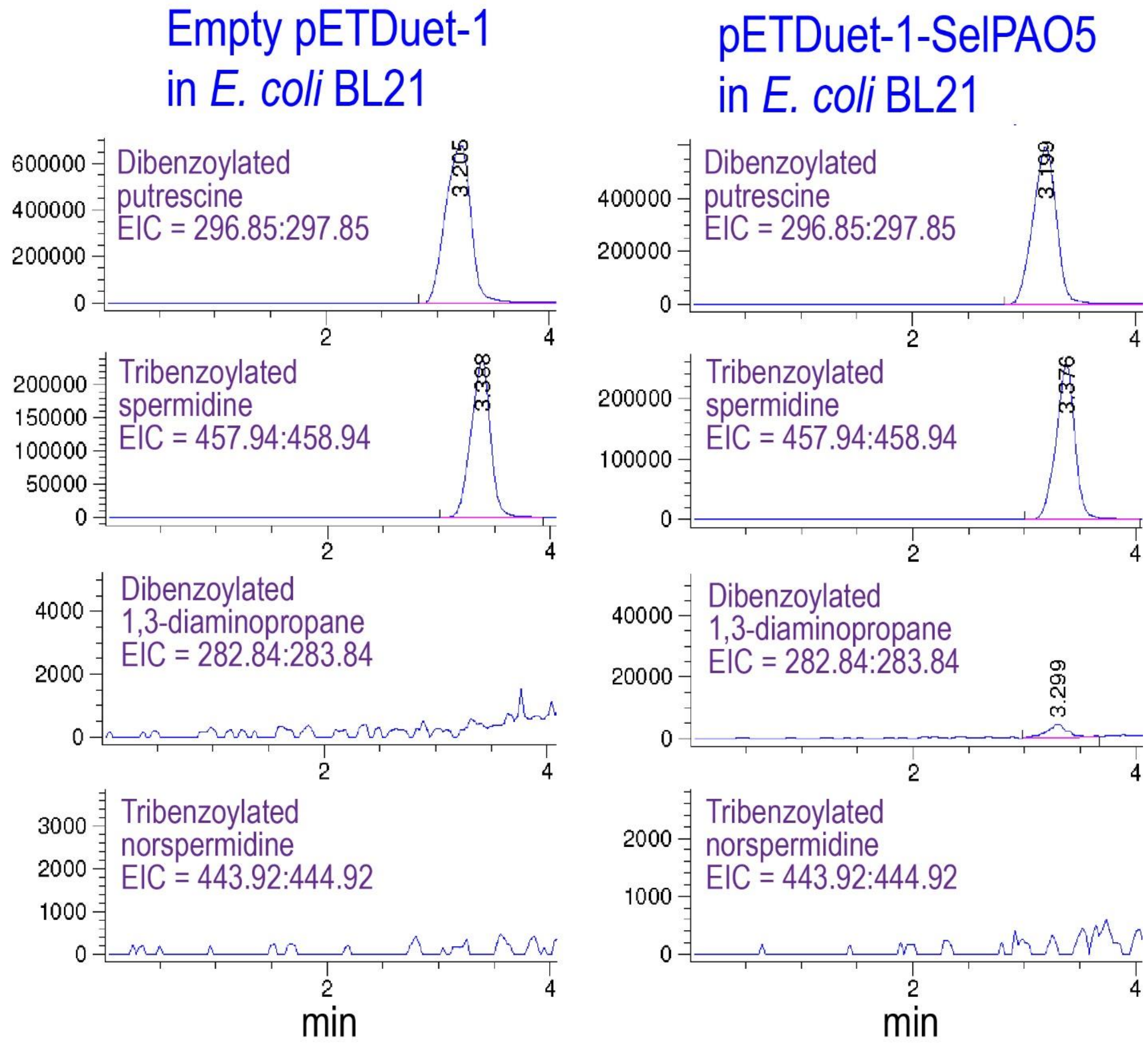

Figure S3. Expression of the *SelPAO5* gene in *E.coli* BL21. Both strains were grown at the same time in polyamine-free M9 medium, and analyzed together. The y-axis represents arbitrary units of ion intensity.
